# Supplementary material for: The sensitivity of magnetic particle imaging and fluorine-19 magnetic resonance imaging for cell tracking
Source: Sci Rep. 2021 Nov 12;11:22198. doi: 10.1038/s41598-021-01642-3 (PMC8589965; doi:10.1038/s41598-021-01642-3)
Supplement: Supplementary file 1 — Supplementary Information. [file 41598_2021_1642_MOESM1_ESM.pdf]

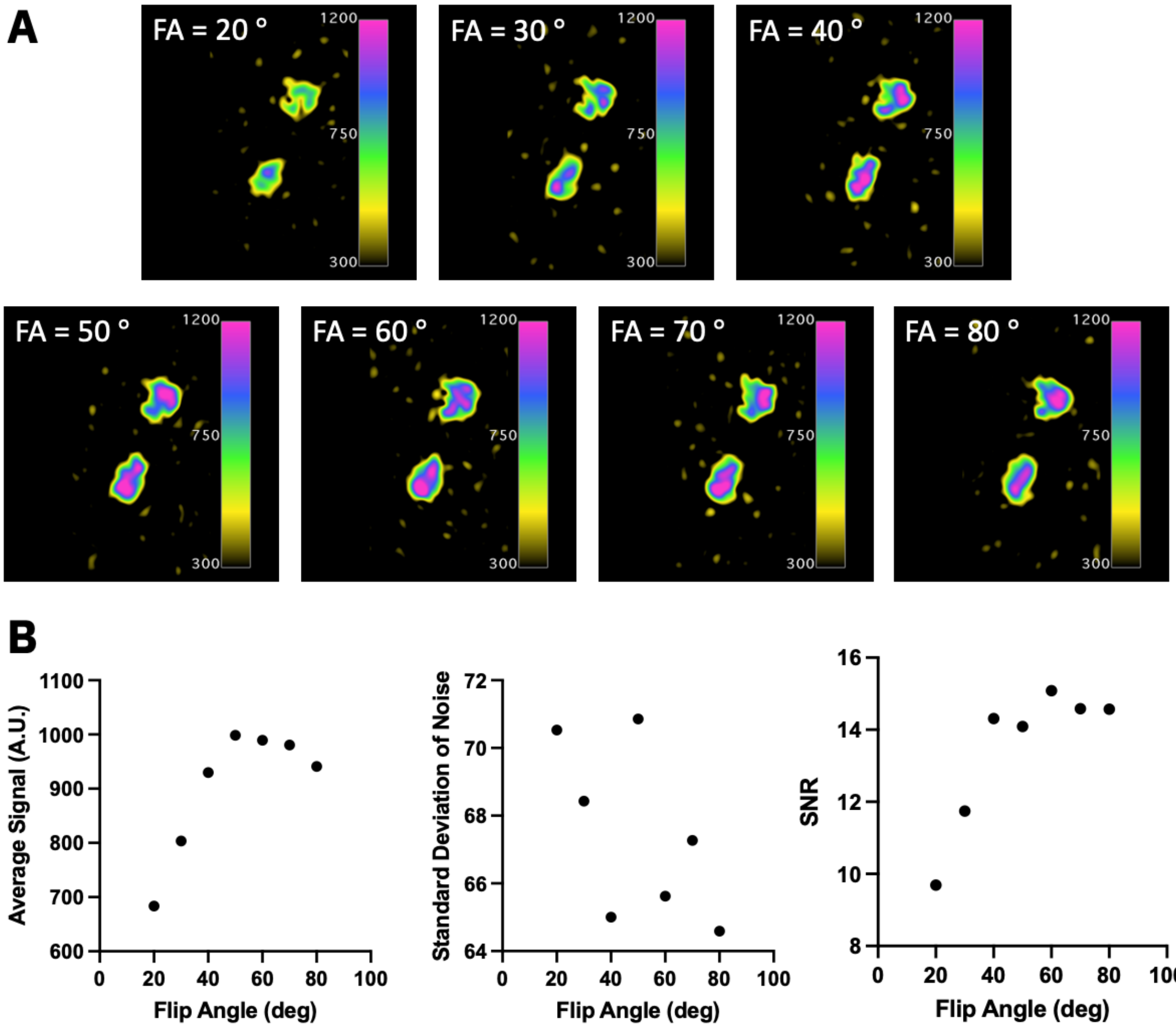

Supplementary Figure 1. Optimization of ^{19}F flip angle at 3 T. (A) Two reference phantoms (3.33 x 10^{16} ^{19}F spins/μL) were imaged using 3D bSSFP with 80 excitations and various flip angles: 20, 30, 40, 50, 60, 70, and 80 degrees. **(B)** The average signal of the reference tubes, the standard deviation of background noise, and the SNR were measured for each flip angle.

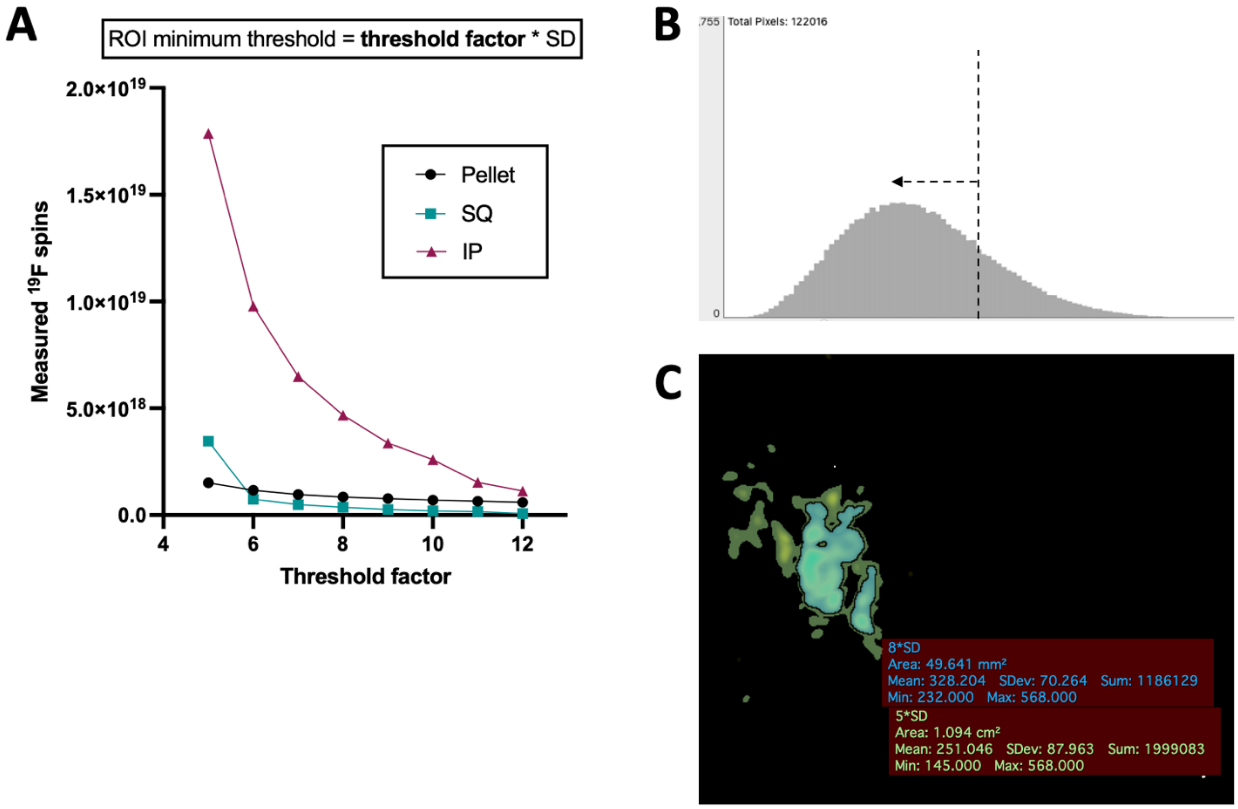

Supplementary Figure 2. (A) Various thresholds ($5*SD_{noise} – 12*SD_{noise}$) were used to segment ^{19}F signal from 2 x 10^6 PFPE-labeled MSC, in a pellet or *in vivo* (subcutaneous, SQ; intraperitoneal; IP). These images are shown in Figure 7. Regardless of the threshold value used, ^{19}F signal measured from cells administered IP exceeded that of SQ and from pellets. In this quantification there is no correction applied to signal values. **(B)** Voxel distribution of background ^{19}F noise was non-Gaussian and left-skewed, indicating there is a Rician distribution of noise. **(C)** A demonstration of the segmentation method described in Bouchlaka et al. (2016)[14] from a single coronal slice of the mouse administered PFPE-cells IP (also shown in **Figure 7B**). ^{19}F signal between $5*SD_{noise}$ – $8*SD_{noise}$ (green) was corrected with a factor of 0.655 and ^{19}F signal exceeding $8*SD_{noise}$ (blue) was not corrected. Signal above $5*SD_{noise}$ was included for ^{19}F quantification.

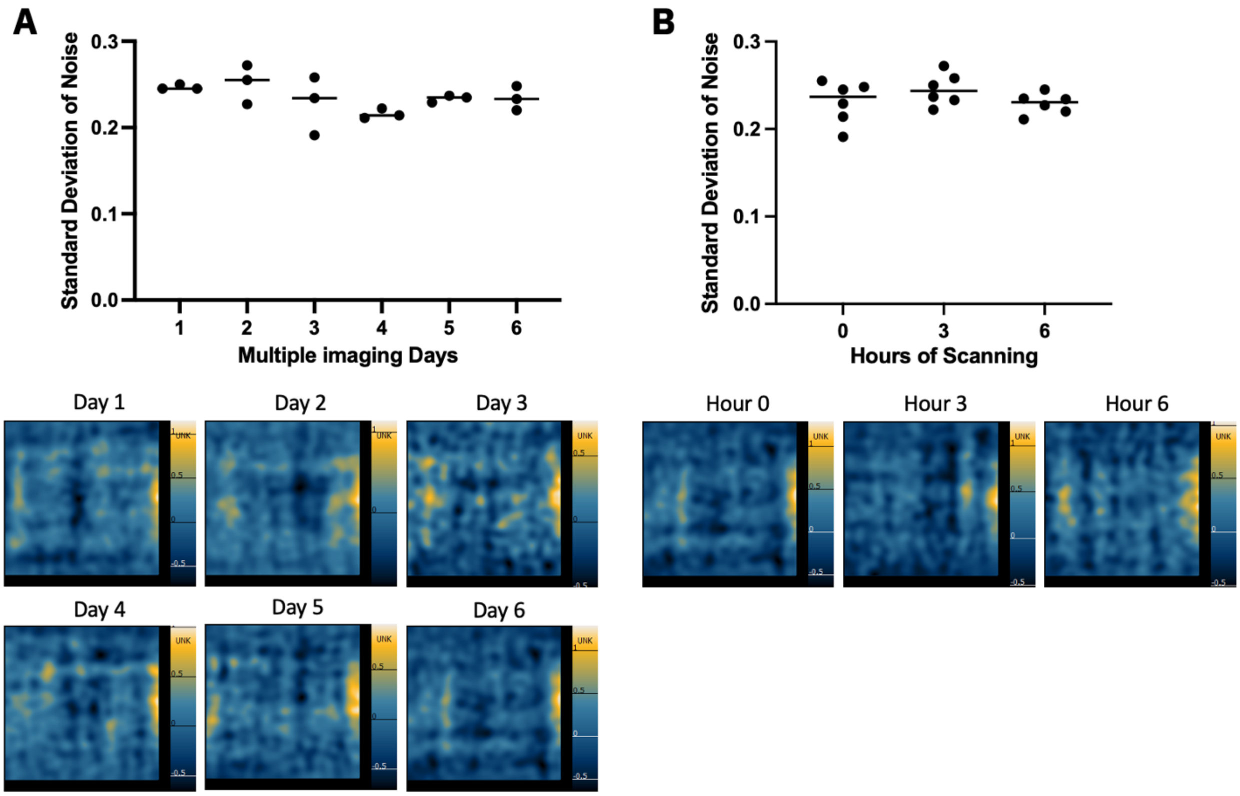

Supplementary Figure 3. The standard deviation of background noise (SD_{noise}) for MPI was measured from an empty sample holder at the beginning (t = 0h), middle (t = 3h), and end (t = 6h) of six imaging sessions. There were no significant differences in measured SD_{noise} between **(A)** imaging days or **(B)** the 3 timepoints.
